# An Acrostic

**Published:** 1887-03-05

**Authors:** 


### AN ACROSTIC.

**H** ere is the place where there's every kind
**O** f help. A restful bed,
**S** urgeon and nurse ready to bind,
**P** erhaps an arm or head,
**I** n here, physicians now at hand
**T** o tend the sick are found.
**A** ll these, a very noble band,
**L** ike angels life surround.—H. J. C.

